# PROTOCOL: Multifaceted interventions for supporting community participation among adults with disabilities: a systematic review

**DOI:** 10.1002/CL2.214

**Published:** 2018-03-22

**Authors:** Judith M.S. Gross, Amalia Monroe‐Gulick, Debbie Davidson‐Gibbs, Chad Nye

## Background

### The problem, condition or issue

The University of Kansas, in partnership with the University of Montana, operates a Rehabilitation Research and Training Center on Promoting Interventions for Community Living (RRTC/PICL). The purpose of the Center is to examine the effectiveness of interventions to support greater community participation for individuals with physical and co‐occurring multiple disabilities and to promote the dissemination and utilization of effective interventions. Currently, in its first year of operation, the RRTC/PICL is charged with developing and promoting interventions that support individuals with disabilities and positively impact community participation outcomes for such individuals. One of the goals of RRTC/PICL is to conduct a systematic review of the literature to identify the outcomes of effective evidence‐based, multifaceted interventions (see page 2 for key definitions) that support individuals with disabilities to participate in their community through.

Numerous U.S. laws have been passed to increase protections and opportunities for people with disabilities to live more independently in the community. Section 504 of the Rehabilitation Act of 1973 was created to increase opportunity for physical and programmatic access in institutions (e.g., universities) that received federal monies. Title VII of the Rehabilitation Act established Centers for Independent Living (CILs), and mandated four core services: information and referral, advocacy, peer counselling, and independent living skills training; a fifth core service, transition, was added in the latest version of the Workforce Innovation and Opportunity Act of 2014 (Administration for Community Living [ACL], 2016). The Fair Housing Amendments Act of 1988 allowed tenants to make reasonable modifications to their apartments at their own expense; the Americans with Disabilities Act of 1990 was designed to protect the civil rights of citizens with disabilities; the Olmstead v. L.C. Supreme Court decision (1999) allowed people to live in least restrictive environments; and the currently proposed Disability Integration Act (DIA), S. 2427 would create a civil right to home and community‐based services and supports as an alternative to institutions. Despite these enacted laws, many people with disabilities lack personal knowledge, skills, resources and/or enabling environments that would allow them to take advantage of these rights and resources and live more independently in the community.

### The intervention

For this review, we rely heavily on research conducted by our research team member Bryce Ward, Ph.D., the Associate Director at the Bureau of Business and Economic Research and Director of the Bureau's Health Care Research Program at the University of Montana. Dr. Ward has published work on community participation of disabled persons in peer‐reviewed journals.

The systematic review is proposed for multifaceted interventions that promote community participation among people with disabilities. A ***multifaceted intervention*** is defined assomething that is done to, with, or for the person with a ***disability*** or the environment in which they interact or want to interact to address two or more individual or environmental characteristics (in different life domains) to achieve ***community participation outcomes***. This is a “broad stroke” definition for the focus of our meta‐analysis. However, the ***bold italicized*** words within this definition are clearly defined below. Through a careful application of these definitions, the team will come to consensus and determine whether an article would meet inclusionary criteria.

**Multifaceted interventions** ‐ address two or more individual (changing something about the person, including enhancing skills/knowledge, changing behaviour, changing perceptions/attitudes) or environmental characteristics (changing something about the people, places, or things in the environments in which the person interacts) in different domains (e.g., social skills/inclusion, financial resources/management, physical health, mental health, employment, transportation, adult learning, health care)

**Disability** – any physical or cognitive limitations, including multiple disabilities

**Community ‐** settings that are integrated with people without disabilities in an environment where people without disabilities typically work, live, and recreate

**Community participation outcomes** –are chosen by/desired by the individual with a disability, occur in the ***community*** (i.e., integrated with people without disabilities),and are reflective of (a) direct access to or participation in the community or (b) dimensions of community participation (i.e., outcomes for which there is a research base linking them to community participation). These are to include the following outcomes:

*Direct access to or participation in the community*

- Integrated competitive employment (i.e., employment in the community with non‐disabled peers for minimum wage or higher)
- Continued learning (e.g., engaging in college or technical training – classes or diploma or certificate completion)
- Housing (e.g., independent living in a home of choice, usability of the home)
- Civic involvement (e.g., voting, volunteer work, advocacy, committees/leadership)
- Recreation (e.g., sports, art, music, community events)
- Navigating the community/accessing community (e.g., accessing public transportation)

*Dimension of community participation*

- Increased self‐determination (e.g., autonomy, decision‐making, self‐advocacy)
- Improved physical or mental health
- Improved general quality of life
- Increased family support/activities in the home (e.g., caregiving, supporting children/parenting, household chores/care)
- Social networking (e.g., friendships, relationships – boy/girlfriend or spouse, church)

We recognize that the nature of the community participation outcomes defined is broad.

Our goal in conducting a systematic review of multifaceted interventions is to identify the current status of multifaceted intervention research for community participation outcomes, point to gaps in the literature on such interventions and features that are effective in enhancing community participation.

Examples of multifaceted interventions resulting in community participation outcomes include the following:

1. A vocational services agency decides to implement customized employment services and social skills training in an effort to affect rates of obtaining and maintaining integrated competitive employment.
2. An independent living center implements an adaptive recreation program at the local community center along with transportation training in the community in an effort to affect the rate at which individuals with physical disabilities are accessing and navigating their community and engaging in recreational activities.

### How the intervention might work

**Figure 1:**
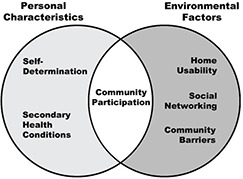
Adapted person‐environment fit model with examples of personal characteristics and environmental factors

The concept of person‐environment fit is the theoretical framework that will guide our work on the systematic review of literature related to community participation interventions. The Person‐Environment diagram (see Figure 1), adapted from Tang et al. (2011), depicts the interaction of personal and environmental factors which adapts existing evidence‐based interventions related to community participation.

The social‐ecological framework meshes with the Institute of Medicine's model of disability as a dynamic process that results from characteristics and interactions of the individual with his/her environment (Brandt Jr & Pope, 1997; Field &Jette, 2007; McDermott & Turk, 2011; Nagi, 1991). “Disability” itself “depends on an interaction between the individual and the physical and social environment” (Rehabilitation Act (Sec. 2(a)(3)). Person‐environment fit theory has been applied in numerous areas, including employee attitudes and behaviors (Duffy, Autin, &Bott, 2015), functioning among older adults in long‐term care settings (Rantakokko, Törmäkangas, Rantanen, Haak, &Iwarsson, 2013), and transition of youth with disabilities to adulthood (Stewart et al., 2014).

In addition to our theoretical orientation, we subscribe to the basic values of Independent Living Philosophy (DeJong, 1978; Pfeiffer, 2002; White, Lloyd Simpson, Gonda, Ravesloot, & Coble, 2010). These values are consistent with the basic guidelines for quality Home and Community‐Based Services (HCBS) related to the provision of Medicaid services to people with disabilities in the community (National Quality Forum, 2015), and provide the ethical and social guidelines for intervention research that go beyond empirically derived best practices. They include, among others, services provided to maximize integration and inclusion in the community; enabling self‐direction, choice, and control; adherence to human and legal rights to personal freedom, privacy, and risk balanced with safety and support; and delivering and managing services that are coordinated, dependable, and comprehensive (National Quality Forum, 2015).

Considering this theoretical and value‐based orientation, we will review studies that take into account various personal characteristics as well as environmental factors. Personal characteristics are often the target of interventions seeking to change something about the person (how they think, what they know, how they act). Environmental factors may be the target of interventions seeking to increase access and opportunity for people with disabilities. We will review studies of interventions that address two or more of these characteristics and factors across different domains and examine the impacts of the intervention on community participation outcomes for a diverse group of people with disabilities.

### Why it is important to do the review

Studies of the impact of single‐faceted interventions on community participation of adults with disabilities are numerous. An example of a systematic review of a single intervention is a study of the efficacy of using service dogs for people with physical disabilities (Winkle, Crowe, & Hendrix, 2012). Other reports examining the impact of a single intervention on community participation for adults with disabilities include a study of transportation vouchers (Samuel, Lacey, Giertz, Hobden, & LeRoy, 2013), an exercise intervention (Dean et al., 2012), and a cognitive intervention to reduce fear of falling and associated avoidance of activity among older adults with disabilities (Haastregt et al., 2007). However, our theoretical orientation is on reviewing the effectiveness of multifaceted interventions, which are defined as interventions that address two or more barriers to change (Eldh&Wallin, 2015). The hypothesis is that multifaceted interventions will be more effective, especially when confronted with multiple, complex, and interacting factors affecting outcomes of a complex phenomenon such as community participation by people with a wide range of disabilities.

There are very few systematic reviews of multifaceted interventions related to disability issues. Most reviews on the topic of disability and community relate to descriptive factors serving as barriers or facilitators to participation (e.g., White et al., 2011). In searching The Cochrane Collaborative, The Campbell Collaboration, and NARIC databases, we found no systematic reviews related to multifaceted interventions to enhance community participation by adults with disabilities of any type. There were a few systematic reviews of multifaceted interventions focused on other topics. For example, Williams et al. (2007) conducted a systematic review of multifaceted interventions to improve depression care and found strong evidence for the effectiveness of care management for depression.

The research question guiding our meta‐analysis is as follows:

- What are the reported community participation outcomes of multifaceted interventions targeting adults with disabilities?

Our findings will be used to enhance the planned intervention research in years 2‐5 of our NIDILRR grant award. The figure below demonstrates the role the systematic review will play in our future grant activities.

**Figure 2:**
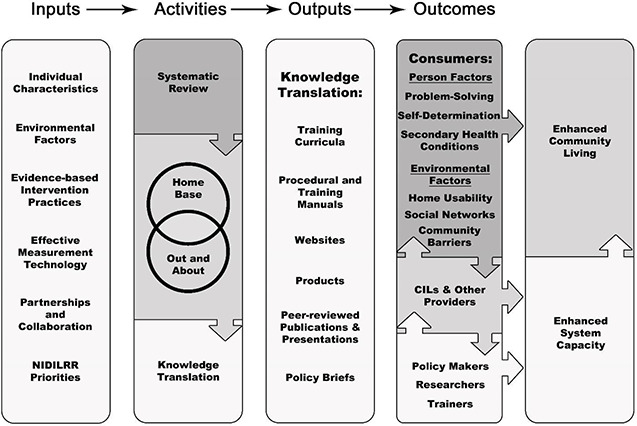
RRTC/PICL Logic Model

## Objectives

The purpose of this systematic literature review is to synthesize the research on the effects of multifaceted interventions provided in community settings for the purpose of promoting community participation outcomes of adults (18+ years old, no longer receiving secondary education services) with disabilities in studies using quantitative – randomized‐controlled trials and quasi‐experimentalresearch design methods.

We seek to identify the outcomes of effective multifaceted interventions that facilitate community participation for adults with disabilities, particularly those with severe limitations (e.g., cognition, mobility) that impede their participation in the community. The results of the literature review will inform policy makers in their practical decisions about social and behavioural interventions and public policy regarding funding and services to provide such interventions. In addition, the literature review results have the potential to help health and social services practitioners in the field who work directly with people with disabilities to understand and apply the information regarding multifaceted interventions in their daily work.

The findings will also inform modifications of our planned interventions to be addressed in the subsequent years of the Center and will be the basis for at least one peer‐reviewed publication. In addition to filling a critical gap in the research literature, we expect the results of the systematic literature review to provide us with information to modify and finalize our multifaceted intervention research.

## Methodology

### Criteria for including and excluding studies

#### Types of study designs

Types of intervention studies that will be reviewed include:

- quantitative – randomized‐controlled trials
- quasi‐experimental
- mixed methods (quantitative component will be included when it meets quality expectations for the methodology)

The team will ensure that the research of selected studies is high‐quality using adapted versions of the National Technical Assistance Center on Transition (NTACT) Quality Indicator Checklists for Group Experimental studies (see http://www.transitionta.org/effectivepractices). The quality indicator checklist for group experimental studies distinguishes between rigorous and weak study designs and identifies a number of quality indicators related to participants, intervention and comparison conditions, outcome measures, and data analysis. Among the 19 indicators, the checklist identifies that studies of high quality meet all but one indicator (#19), and acceptable quality studies meet 10 specific indicators out of the 19. Studies not meeting those 10 specific indicators are considered to be of unacceptable, or weak, methodological quality and will be excluded from the sample. We will include all studies meeting “acceptable” and “high quality” standards as indicated by the application of the checklist. Adaptations to the checklist include removing any requirement that study content be specific to the field of transition to adulthood.

Types of intervention studies that will not be included in the review:

- case studies
- single subject research design
- single group – pre/post

*Inclusionary and exclusionary criteria*

Inclusionary criteria:

- Published 2000‐present
- Peer reviewed – including published work and dissertations/theses
  ∘ This meta‐analysis was undertaken as a component of a research agenda for the federally funded Research and Training Center on Promoting Interventions for Community Living (RRTC/PICL). In the funded grant proposal, one research activity to be completed for the purposes of informing future research at the RRTC/PICL was the completion of a systematic review of peer‐reviewed literature on multifaceted interventions promoting community participation. In addition, since the topic is expansive, covering multiple types of interventions with a wide array of possible outcomes, we expect there to be quite a large number of search results that will need reviewed for inclusion.
- Original research involving testing an intervention
  ∘ Intervention has a clearly defined disability sample
    ■ It is the entire sample or a subsection of the entire sample that is receiving the intervention with outcomes identified specific to that sample.
  ∘ Participants are 18+ years of age and have left secondary education
  ∘ Intervention is applied in a community‐based setting
  ∘ Applied intervention is multifaceted
- Measures of community participation outcomes for the disability sample
- English only
  ∘ We specifically are working with English only studies because the concepts and terminology we are using varies from culture to culture. We have struggled to clearly define terms within English for our work as things like “community‐based” and “community participation” may be different from culture to culture and even terms in English that define disability vary from culture to culture. While our research group has broad and varied skillsets, it is difficult to come to agreement among people who lack the same disability background and historical knowledge of disability in the culture of study. We also are trying to define “multifaceted interventions” and identify a base of intervention studies that reflect that definition. Considering these challenges, we feel that limiting our studies to English language only will help to ensure reliability in the application of the inclusionary criteria.

Exclusionary criteria:

- Articles that are a literature review or compilation of studies (may use these for bibliographic searches for primary research articles)
- Intervention studies applied in segregated environmentsin which the only people without disabilities are those in a service or leadership capacity serving/supporting the adults with disabilities

#### Types of participants

The intervention must target persons with a disability/ies for at least a portion of the target sample, with outcomes identified specific to the disability sample. This means that the intervention must be provided to individuals with disabilities, whether they represent the entire sample in the study or a subsection of the sample. The outcomes for the individuals with disabilities must be analysed separate from individuals in the sample without a disability.

Samples in selected articles will include adults:

- Who are 18+ years old
- Whom have left the secondary education/high school setting
- Whom have one or more disabilities

Study samples will **not** include individuals under 18 years old or who are still participating in a transition program (18‐22 years) in secondary special education. All other multifaceted interventions targeting increasing community participation of adults with disabilities will be included in the search.

#### Types of interventions

The intervention in the study must meet the following criteria:

- Intervention measures one or more community participation outcomes targeted for change
- Intervention targets persons with a disability/ies for at least a portion of the sample – with an identifiable impact on the disability sample
- Intervention seeks to change/impact two or more personal characteristics or environmental factors that are in different domains (e.g., transportation skills and employability skills)

See examples of multifaceted interventions are provided on page 4.

#### Types of outcome measures

Community participation outcomes are chosen by/desired by the individual with a disability, occur in the community (i.e., integrated with people without disabilities), and are reflective of (a) direct access to or participation in the community or (b) dimensions of community participation (i.e., outcomes for which there is a research base linking them to community participation). Below we have provided examples of what might be included in each broad outcome category. However, we do not want to limit our community participation outcomes by strictly defining each term below.

These are to include the following outcomes and examples below:

*Direct access to or participation in the community*

- Integrated competitive employment
- Continued learning
  ∘ Education
  ∘ Training
- Housing
  ∘ Place
  ∘ Housemates
  ∘ Usability
- Civic involvement
  ∘ Voting
  ∘ Advocacy
  ∘ Committees/leadership
  ∘ Volunteer work
- Recreation
  ∘ Sports
  ∘ Arts
  ∘ Music
  ∘ Community events (e.g., art in the park, parades, block parties)
- Navigating the community/accessing community

*Dimension of community participation*

- Increased self‐determination
  ∘ Autonomy
  ∘ Self‐advocacy
- Improved health
  ∘ Physical
  ∘ Mental
- Improved quality of life
- Increased family support/activities in the home
  ∘ Caregiving
  ∘ Supporting children/parenting
  ∘ Household chores/care
- Social networking
  ∘ Friendships
  ∘ Relationships (e.g., boy/girlfriend, spouse)
  ∘ Church/religious activities

Descriptive studies that do not include outcome measures will be excluded. Outcome measures that may be included in the meta‐analysis are outcomes of the individual with a disability only. These may be performance measures, self‐reported, observed and recorded, or caregiver (e.g., family or staff member) reported outcomes measures. We will not include data on outcomes for individuals other than the person with a disability in the meta‐analysis.

#### Duration of follow‐up

All studies meeting the inclusionary criteria and reporting follow‐up data will be analysed for treatment effects for the key outcome variables of interest. The follow‐up conditions will use the same criteria and statistical procedures as the primary intervention data and be reported separately from the primary intervention data.

#### Types of settings

- The intervention must be applied in a community‐based setting.
- Interventions conducted inin‐patient institutions or hospitals will be excluded.

### Search strategy

Our team has the support of a University of Kansas Associate Faculty Librarian with experience on conducting systematic and scoping reviews (Peterson‐Besse et al., 2014; White et al., 2013). Database selection is important because of the need to have the most comprehensive, but least duplicative results as possible. Therefore, the first step is to utilize relevant articles and journals in which the researchers and advisory team predict the most relevant studies would appear, and then identify which electronic databases index the journals by searching Ulrich's Periodical database. The previously identified articles will also inform the initial development of the database search strategy. We will search the databases with most number of journals indexed, while also ensuring that all identified journals are captured. After reviewing fifteen databases and one publisher journal package, we selected three databases for initial peer‐reviewed literature electronic searching: PubMed, Web of Science, and PsycInfo.

The databases were selected based guidelines in the Cochrane Handbook about database selection, including the necessity of searching of PubMed, and the inclusion of both a general bibliographic and a subject‐specific database (Higgins & Green, 2011). The search strategies are informed by previous systematic reviews (Peterson‐Besse et al., 2014; White et al., 2013). The goal is to ensure that all types of disabilities are included in the search results, but also exclude irrelevant results. In addition, we will consider that each database has a specific search strategy because of the different controlled vocabulary and search mechanisms.

Two additional databases, Dissertations & and Theses Abstracts and PolicyFile, will be searched to find potentially relevant grey literature. These two databases were selected because of the variety literature offered, as well as the controlled searching environment. PolicyFile's sources includes think tanks, universities, publishers, and non‐profits, both domestic and international. Dissertation and Theses Abstracts also provides potentially more current and comprehensive research than the peer‐reviewed literature.

Mesh terms will be used in PubMed, however, the “exploding” command except for “disabled persons” and “mentally disabled person,” will not be utilized. In test searches, the “explode” command retrieves excessive numbers of irrelevant and redundant searches; therefore, using the “exact” command establishes a more effective strategy. In the PyscInfo search, “mentally ill” is not included because the preferred term is “mental disorder.” Unlike PubMed and PsycInfo, Web of Science does not have a controlled vocabulary; therefore, searching operators search “near” must be utilized to establish an accurate, but complete search. Web of Science also requires extensive use of “Web of Science Categories” and “Research Areas” as limiters because of the lack of controlled vocabulary. Two members of the research team based on the search results will manually select these. However, similar subject limiters in PubMed and PsycInfo will also be utilized, even though this is not standard protocol. This review search is very broad, specifically the intervention portion of the search, and the number of results will be too large without the use of database provided subject and/or classification limiters. The results from all three searches will be combined, exported, and reduplicated using the reference management software EndNote.

We will also develop a spreadsheet with terms related to persons with disabilities that are likely to appear in applicable studies (e.g., sensory disorders, hearing loss, dependent amputation mobility limitation, vision disorders).

**Key words to search by include (see** Appendix A **for detailed search strategy):**

- Community
  ∘ Participation
  ∘ Living
  ∘ Access
  ∘ Community‐based
- Intervention
- Training
- Multifaceted
- Supports and services
- Intervention outcomes (see list in definitions for outcomes key words)

In addition, we identified fields of research where we might find literature and journals associated with each topic as follows.

- Employment & Rehabilitation
  ∘ Disability and Rehabilitation
  ∘ Journal of Vocational Rehabilitation
  ∘ Journal of Applied Rehabilitation Counseling
  ∘ Rehabilitation Research, Policy, and Education
  ∘ Journal of Occupational Rehabilitation
  ∘ Journal of Labor Policy
  ∘ Journal of Rehabilitation
  ∘ Rehabilitation Counseling Bulletin
- Community psychology
  ∘ Journal of Counseling Psychology
- Medicine/public health
  ∘ American Journal of Public Health
- Aging
  ∘ The Gerontologist
- Disability
  ∘ Disability and Society
  ∘ Research in Developmental Disabilities
  ∘ Intellectual and Developmental Disabilities
  ∘ Disability and Health Journal
  ∘ Journal of Intellectual and Developmental Disability
  ∘ Journal of Intellectual Disabilities
  ∘ Journal of Applied Research in Intellectual Disabilities
  ∘ Autism
  ∘ Journal of Intellectual Disability Research
  ∘ Journal of Disability Policy Studies
  ∘ Research and Practice for Persons with Severe Disabilities
  ∘ Focus on Autism and Developmental Disabilities
  ∘ Education and Training in Developmental Disabilities

### Description of methods used in primary research

Anticipated methods that studies are likely to employ include random assignment to treatment and control/comparison conditions or pre‐test/post‐test designs. We expect that the treatment conditions will be compared to either a no‐treatment control group or a comparison group receiving a different intervention. Studies will be subjected to two levels of analysis to generate an overall study effect size controlling for such variables as design (e.g., random vs quasi‐experimental), outcome (e.g., competitive employment), participant (e.g., Physical vs Intellectual Disability), or treatment (e.g., individual vs environmental) characteristics.

### Criteria for determination of independent findings

The independence of study findings will be accounted for by insuring that all outcome aggregation will be conducted only for those outcomes of the same conceptual construct, that studies generating outcome data using the same participants will be restricted to a single data set for any sample of participants, and multiple sets of the same data from a participant sample will not be included in any analysis. All meta‐analyses will aggregate outcome results according to common outcome construct and assessment time point to account for the independence of the individual study effect size calculations.

### Details of study coding categories

Coders will engage in preliminary practice using the inclusionary/exclusionary criteria. Coders will each read, review, and discuss articles to test the clarity of the inclusionary criteria. During this process, criteria, definitions, and examples will be clarified. Coders will then apply these criteria to a set of practice articles, reviewing them independently and then discussing and reviewing the application of the criteria – clarifying criteria as needed. Practice sets will be continued until coders reach 80% reliability with regard to screening of practice sets for all inclusionary criteria – editing and enhancing definitions as needed. Once 80% or better is achieved in practice articles, we will conduct the first stage of review.

The first stage of applying inclusionary criteria will include a review of the title and abstract of all searched articles to determine if they can be excluded based on the inclusionary and exclusionary criteria. This first stage of review will be conducted by three members of the research team. Any articles where it is unclear whether or not they would be included will be set aside for a more detailed review of the PDF to determine appropriateness for inclusion in the review.

The second stage of applying the inclusionary criteria involves a review of the PDF. Coding for all included studies will be accomplished by at least two independent reviewers completing a coding sheet in Excel. We will develop a coding sheet to track the decisions for each criteria as well as final decisions for determination of inclusion in the next phase of review. Prior to independently reviewing articles, we will achieve 80% interrater reliability. The application of the criteria and decisions will be discussed whenever there are disagreements regarding the inclusion of the article. We will come to consensus on each decision regarding inclusion.

The third and final stage of review will be conducted regarding the quality of the studies. We will use an adapted versions of the National Technical Assistance Center on Transition (NTACT) Quality Indicator Checklists for Group Experimental studies (see http://www.transitionta.org/effectivepractices). We will insert the criteria into an excel sheet and to track the decisions for each criterion as well as final decisions for determination of inclusion in the review. The application of the criteria and decisions for each article in this stage will be discussed among the 2‐3 reviewers that we expect to be involved in this stage. Whenever there are disagreements regarding the inclusion of the article, we will discuss the issues and come to consensus on each decision regarding inclusion.

### Statistical procedures and conventions

Quality Assessment

Each included study will be assessed for methodological quality using as the risk of bias for such characteristics as reporting, internal validity bias, selection bias, and external validity bias attrition bias using the National Technical Assistance Center on Transition. (see Appendix A). The quality assessment will follow the same review approach used for determining study inclusion using two independent reviewers as described earlier.

Effect Size Calculation and Interpretation

All calculations will be conducted using Comprehensive Meta‐Analysis (CMA) software to synthesize, compute, compare, and determine variation in effect sizes and treatment effects across studies that are reviewed. The calculation of effect sizes will take into account the variety of measures used to assess intervention effects by calculating and analysing all data using the standardized mean difference (SMD) converted to Hedges’ *g* to account for small n sample sizes. All effect sizes will be calculated using the 95% confidence interval (CI). It is anticipated that most of the studies will present with continuous data resulting in the reporting of means and standard deviations. For those studies reporting F‐test, t‐test, or p‐values rather than means and standard deviations effect sizes will be calculated using appropriate conversion conventions provided by CMA. Should a study present report outcomes using dichotomous data, we will calculate the appropriate odds ratio (OR) or risk ratio (RR) and convert the values to Hedges’ *g*.

In order to provide a substantive interpretation of the intervention effects and retain effect size dependence, the effect sizes from the individual studies will be aggregated within and across studies using only same or conceptually similar outcomes. For example, those studies reporting data for hours worked, salary, job tenure, these will be classified as employment outcomes with the combined treatment effect on employment aggregated across other studies reporting employment outcomes. We anticipate categories of outcomes to include employment, mental health, housing, independent/adult daily living, social, learning, and quality of life. Different categories of outcomes will not be aggregated.

Heterogeneity Analysis

Heterogeneity analysis will be conducted for participant, intervention, and outcome characteristics. In light of the fact that multiple effect sizes may be attributable to sampling error, a random effects model and the associated inverse variance weight at the 95% confidence level will be used for all analysis. The random effects model provides for an assumption of population variation from which the sample is drawn and calculates the impact of the effect size by estimating the parameters of that population. Additionally, the random effects model provides the opportunity to account for studies not included in the current data set thus allowing for generalization beyond the present study.

Sensitivity Analysis

A sensitivity analysis will be conducted using the funnel plot function with the trim and fill analysis to assess the strength of conclusions generated from the analysis for variables such as publication bias, publication source, attrition, or missing data. In addition, we will conduct a sensitivity to assess the impact of outlier data (1) the cumulative treatment effect based on the chronological order of the study's publication, (2) study design on the overall effect size, and (3) any single on the overall effect size. Additionally, a sensitivity analysis will be conducted to assess the impact of any outliers by comparing the average weighted effect size with the outliers present and removed. These results will determine the decision to retain or exclude specific data points or implement a Winzorizing procedure to account for their impact.

Subgroup Analysis

Subgroup analysis will be conducted if the number of studies is sufficient, on moderators such as (1) types of direct access to community participation, (2) dimension of community participation, (3) length of intervention, (4) place of intervention, (5) type of outcome measure, and (6) disability. Subgroup analysis will be conducted using CMA.

Missing Data

Attrition will be calculated for each study and a sensitivity analysis conducted to assess effect size impact.

### Treatment of qualitative research

Qualitative studies will be excluded from analysis.

### Review authors

**Lead review author:** The lead author is the person who develops and coordinates the review team, discusses and assigns roles for individual members of the review team, liaises with the editorial base and takes responsibility for the on‐going updates of the review.

**Table:** 

Name:	Judith M.S. Gross
Title:	Center Director
Affiliation:	Indiana University
Address:	1905 North Range Road
City, State, Province or County:	Bloomington, IN
Post code:	47408
Country:	USA
Phone:	812‐855‐7484
Email:	jmsgross@iu.edu
**Co‐author(s):**	
Name:	Amalia Monroe‐Gulick
Title:	Associate Librarian
Affiliation:	University of Kansas
Address:	1425 Jayhawk Blvd.
City, State, Province or County:	Lawrence, KS
Post code:	66045
Country:	USA
Phone:	785‐864‐3377
Email:	almonroe@ku.edu
	
Name:	Debbie Davidson‐Gibbs
Title:	Researcher
Affiliation:	American Institutes for Research
Address:	1000 Thomas Jefferson Street, NW
City, State, Province or County:	Washington, DC
Post code:	20007‐3835
Country:	USA
Phone:	202 403‐6216
Email:	ddavidson-gibbs@air.org
	
Name:	Chad Nye
Title:	Consultant
Affiliation:	American Institutes for Research
Address:	1931 Birchwood Loop
City, State, Province or County:	Lakeland, FL
Post code:	33811
Country:	USA
Phone:	407‐496‐8357
Email:	chadnye@gmail.com

### Roles and responsibilities

- Content:
  ∘ Judith Gross– Judith has worked in the field of special education for over 25 years. She has experience and education that is cross disability and lifespan. She has 12 years of experience conducting training, technical assistance, and research in areas related to adults with disabilities and community participation, especially with regard to integrated and competitive employment.
  ∘ A Scientist‐Consumer Advisory Panel (SCAP) has been established for our broad research which includes the systematic literature review. The role of the Systematic Review SCAP subcommittee is to offer guidance on selection of keywords, inclusion / exclusion criteria, and quality standards and to advise staff on specific issues that arise in conducting the literature review. At the conclusion of the systematic literature review on multifaceted interventions related to community participation, we will convene a meeting of all SCAP members, including the co‐director from the HCBS RRTC on Outcome Measurement, to discuss the implications of our findings.
- Systematic review methods: Judith Gross, Chad Nye, and Debbie Davidson‐Gibbs
  ∘ Judith Gross–Judith conducted a qualitative systematic literature review for her dissertation related to participant direction of supports and services.
  ∘ Chad Nye – A consultant who has conducted systematic reviews published in Campbell, Cochrane, and refereed publication.
  ∘ Debbie Davidson‐Gibbs – A Researcher with American Institutes for Research (AIR), worked with a team to conduct a systematic literature review for The College Board. In 2016, she participated in an 8‐hour workshop sponsored by AIR on conducting Systematic Literature Reviews. In 2017, Debbie participated in a webinar sponsored by QSR International titled: *Accelerating Your Literature Review with NVivo 11 for Windows*.
  ∘ Amalia Monroe‐Gulick ‐ University of Kansas Associate Faculty Librarian with experience conducting systematic and scoping reviews (Peterson‐Besse et al., 2014; White et al., 2013). She has extensive training in: database searching and retrieval, online database interfaces, quantitative and qualitative research methods, and systematic reviews.
- Statistical analysis: Chad Nye– experience using CMA
- Information retrieval: Amalia Monroe‐Gulick is an Associate Librarian at KU who has previously worked with the RRTC/Community Living on two different systematic reviews. MLS, Indiana University; MS, Political Science, Illinois State University; BS, Political Science, Illinois State University

### Sources of support

This systematic review will be conducted under a grant from the National Institute on Disability, Independent Living, and Rehabilitation Research (NIDILRR grant number 90RT5043‐01‐00). NIDILRR is a Center within the Administration for Community Living (ACL), Department of Health and Human Services (HHS). The contents of this systematic review do not necessarily represent the policy of NIDILRR, ACL, HHS, and you should not assume endorsement by the Federal Government.

In addition, AIR provided staffing support (research assistant and consultant) and technical assistance in the completion of the review to meet Campbell standards.

### Declarations of interest

There are currently no known conflicts of interest. However, it is possible that articles by White, Ravesloot, Summers, or Nary (project staff and research partners) may be selected in the article search process if they meet the inclusionary criteria.

### Preliminary timeframe

Approximate date for submission of the systematic review.

10/31/17

### Plans for updating the review

For the RTC/PICL, we plan to use the completed review to inform design and development of multi‐faceted intervention research in the remaining four years of the Center. Specifically, we have two multi‐faceted intervention research projects currently in the development phase, one involving modifications to the home environment, and the other involving modifications to individual characteristics, including developing problem solving skills, promoting health and well‐being, and building self‐advocacy skills, to support enhanced community participation. Factors identified in the systematic review as most effective in terms of interventions, will be incorporated into these interventions. In addition, our Knowledge Translation project will support continued technical assistance and dissemination of information to consumers wishing to know more about the value of multi‐faceted interventions. Updating the review will be the responsibility of the Research Director, in cooperation with the Dissemination Coordinator.

### AUTHOR DECLARATION

#### Authors’ responsibilities

By completing this form, you accept responsibility for preparing, maintaining and updating the review in accordance with Campbell Collaboration policy. The Campbell Collaboration will provide as much support as possible to assist with the preparation of the review.

A draft review must be submitted to the relevant Coordinating Group within two years of protocol publication. If drafts are not submitted before the agreed deadlines, or if we are unable to contact you for an extended period, the relevant Coordinating Group has the right to de‐register the title or transfer the title to alternative authors. The Coordinating Group also has the right to de‐register or transfer the title if it does not meet the standards of the Coordinating Group and/or the Campbell Collaboration.

You accept responsibility for maintaining the review in light of new evidence, comments and criticisms, and other developments, and updating the review at least once every five years, or, if requested, transferring responsibility for maintaining the review to others as agreed with the Coordinating Group.

#### Publication in the Campbell Library

The support of the Coordinating Group in preparing your review is conditional upon your agreement to publish the protocol, finished review, and subsequent updates in the Campbell Library. The Campbell Collaboration places no restrictions on publication of the findings of a Campbell systematic review in a more abbreviated form as a journal article either before or after the publication of the monograph version in Campbell Systematic Reviews. Some journals, however, have restrictions that preclude publication of findings that have been, or will be, reported elsewhere and authors considering publication in such a journal should be aware of possible conflict with publication of the monograph version in Campbell Systematic Reviews. Publication in a journal after publication or in press status in Campbell Systematic Reviews should acknowledge the Campbell version and include a citation to it. Note that systematic reviews published in Campbell Systematic Reviews and co‐registered with the Cochrane Collaboration may have additional requirements or restrictions for co‐publication. Review authors accept responsibility for meeting any co‐publication requirements.

**I understand the commitment required to undertake a Campbell review, and agree to publish in the Campbell Library. Signed on behalf of the authors:**

**Form completed by: Judith Gross**

**Date:**

## PubMed Search Strategy

1. Mentally Disabled Persons[Mesh]
2. Disabled persons[MesSH]
3. Intellectual Disability[mh:noexp]
4. Vision Disorders[mh:noexp]
5. Communication Disorders[mh:noexp]
6. Visually Impaired Persons [mh:noexp]
7. Amputees[mh:noexp]
8. Mentally Ill Persons [mh:noexp]
9. Persons With Hearing Impairments[mh:noexp]
10. Developmental Disabilities[mh:noexp]
11. Cognition Disorders[mh:noexp]
12. Neurocognitive Disorders[mh:noexp]
13. Mental Disorders[mh:noexp]
14. Mobility Limitation[mh:noexp]
15. Dependent ambulation[mh:noexp]
16. Paraplegia[mh:noexp]
17. quadriplegia[mh:noexp]
18. hearing loss[mh:noexp]
19. blindness[mh:noexp]
20. mental retardation[mh:noexp]
21. Sensory Disorders[mh:noexp]
22. Hearing impairment*
23. Hearing loss*
24. Sensor* disorder*
25. Amputee*
26. Dependent ambulation*
27. Mobility impairment*
28. Activity limitation*
29. Vision disorder*
30. Vision impairment*
31. Participation limitation*
32. Functional limitation*
33. Cognitive impairment*
34. Psychiatric disabilit*
35. Intellectual disability*
36. Mental retard*
37. Or/1‐36
38. Community Integration[Mesh]
39. Education, Nonprofessional[Mesh]
40. Rehabilitation[Mesh:NoExp]
41. Recreation Therapy[Majr]
42. Rehabilitation/education[Mesh]
43. lifestyle intervention
44. Psychotherapy[Mesh]
45. Health Promotion[Majr]
46. Mentoring[Majr]
47. Simulation Training[Mesh:NoExp]
48. Or/38‐48
49. 37 and 49

## PubMed Filters

Publication Years: 2000‐present

Species: Humans

Included study types

Adult Only

English only

## Web of Science Search Strategy

1. disabled
2. developmental near/1 disabilities
3. “communicative disorders”
4. communicative near/1 disorders
5. “mental retardation”
6. paraplegia
7. quadriplegia
8. hearing near/1 disorders
9. deafness
10. hearing near/1 loss
11. vision near/1 disorder
12. blindness
13. disabled near/2 person
14. disabled near/2 people
15. cognitive near/1 impairment
16. visually near/1 impaired
17. vision near/1 impairment
18. developmental NEAR/1 disabilities
19. mentally disabled person*
20. intellectual near/1 disability
21. mobility near/1 limitation
22. mobility near/1 impairment
23. mental near/1 disorders
24. chronic near/1 “mental disorders”
25. psychiatric near/1 disability
26. mentally near/1 ill
27. mental near/1 health near/1 impairment
28. physical near/1 disability
29. Or/1‐28
30. social near/1 support
31. Behavioral near/1 Intervention
32. psychological near/1 intervention
33. training near/1 needs
34. vocational near/1 rehabilitation
35. Vocational and education and training;
36. Home‐based near/1 physical near/1 therapy
37. physical near/1 therapy
38. speech near/1 therapy
39. occupational near/1 therapy
40. psychological near/1 rehabilitation
41. rehabilitation near/1 programs
42. community near/1 based near/1 rehabilitation
43. cognitive near/1 behavioral near/1 therapy
44. supported near/1 employment
45. individual near/1 placement near/1 support
46. health near/1 promotion
47. workshops
48. support near/1 services
49. community near/1 services
50. psychological near/1 therapy
51. Or/30‐50
52. 29 and 51

### Web of Science Filters

**Filters**

Publication Years: 2000‐present

AND

Document Type (Article)

English only

AND

Countries: USA

**Dissertations & and Theses Abstracts Search Strategy**

1. Exact(“developmental disabilities”)
2. Exact(“learning disabilities”)
3. Exact(“reading disabilities”)
4. Exact(“mental disorders”)
5. Exact(“deafness”)
6. Exact(“blindness”)
7. Exact(“quadriplegia”)
8. Exact(“paraplegia”)
9. Exact(“behavior disorders”)
10. Exact(“special needs”)
11. Exact(“hearing impairments”)
12. Exact(“intellectual disability”)
13. Exact(“hearing disorders”)
14. Exact(“vision disorders”)
15. Exact(“Cognitive Impairment”)
16. Exact(“psychiatric patients”)
17. Exact(“mental illness”)
18. all(psychiatric disabilit*)
19. all(mobility impairment*)
20. diskw(disabilit*)
21. or (1‐20)
22. Exact(“intervention”)
23. Exact(“rehabilitation”)
24. Exact(“employment training programs”)
25. Exact(“training”)
26. Exact(“therapy”)
27. diskw(coach*)
28. diskw(workshop*)
29. diskw(intervent*)
30. or (22‐29)
31. 21 and 30

Dissertations & and Theses Abstracts Filters:

Publication Years 2000‐present

And

English only

***PolicyFile Search Strategy***

1. (all(developmental disabilities))
2. (all(learning disabilities))
3. (all(reading disabilities))
4. (all(mental disorders))
5. (all(deaf*))
6. (all(blind*))
7. (all(behavior disorders))
8. (all(special needs))
9. (all(hearing impairment))
10. (all(intellectual disability))
11. (all(hearing disorder))
12. (all(vision disorders))
13. (all(Cognitive Impairment))
14. (psychiatric patients)
15. (all(mental illness))
16. (all(psychiatric disabilit*)
17. (mobility impairment*)
18. (all(disabilit*))
19. or (1‐18)
20. (intervention)
21. (rehabilitation)
22. (employment training programs)
23. (training)
24. (therapy)
25. (coach*)
26. (workshop*)
27. (intervent*)
28. or (20‐27)
29. 19 and 28

PolicyFile Filters

Publication Years: 2000‐Present

And

English only

## System Level Meta‐Analysis Coding Scheme

A. **Bibliographic References**
  1. Study main author:
  2. Title of study:
  3. Year of publication:
  4. Country of study:
  5. What type of publication is the study?
    a. □ Book or book chapter
    b. □ Journal article
    c. □ Thesis or dissertation
    d. □ Technical report (e.g., private report, government report)
    e. □ Conference paper
    f. □ Online document (e.g., Google, website)
    g. □ Other (Specify):
  6. What discipline is the study from?
    a. □ Post‐secondary education
    b. □ Mental health (e.g., psychology, counseling, psychiatry)
    c. □ Nursing/Medicine/Health care
    d. □ Ethnic/multicultural studies
    e. □ Social work
    f. □ Disability rehabilitation
    g. □ Vocational rehabilitation or related employment service system
    h. □ Aging and disability
    i. □ Other (Specify):
B. **Participant Characteristics**
  7. Who was the main recipient of intervention (Treatment Group)? (pg. __)
    a. □ Person with a disability
    b. □ Family member or caregiver
    c. □ Service provider/professional
    d. □ Community member
    e. □ Other (Specify):
  8. From where were the participants drawn? (pg. __)
    a. □ Hospital
    b. □ Outpatient Clinic
    c. □ Educational Organization
    d. □ Community Service Organization
    e. □ Cannot tell/Not reported
    f. □ Other (Specify):
  9. What was the SES of treatment group? (pg. __)
    a. □ Low
    b. □ Middle
    c. □ High
    d. □ Mixed SES
    e. □ Cannot tell/Not reported
  10. What was the race/ethnicity of the treatment group? (pg.)
    a. □ White
    b. □ African American
    c. □ Asian
    d. □ Hispanic/Latino
    e. □ American Indian or Native Alaskan
    f. □ Mixed
    g. □ Other (Specify):
    h. □ Cannot tell/Not reported
  11. What was the predominant disability/health condition of the treatment group? (pg. __)
    a. □ Intellectual and/or developmental disability
    b. □ Hearing impairment
    c. □ Visual impairment
    d. □ Physical disability
    e. □ Traumatic brain injury
    f. □ Mental illness (e.g., schizophrenia, depression, PTSD)
    g. □ Aging disability
    h. □ Cannot tell/Not reported
    i. □ Other (Specify):
C. **Intervention Characteristics**
  12. Where did the intervention received by the whole sample take place? (pg. __)
    a. □ Outpatient hospital or clinic
    b. □ Rehabilitation facility/center
    c. □ Community mental health center
    d. □ Nursing home/assisted living
    e. □ Community disability service organization
    f. □ Home
    g. □ Community at large
    h. □ Cannot tell/Not reported
    i. □ Other(s) (Specify):
      Comments:
  13. Geographical setting of the intervention? (pg.)
    a. □ Urban
    b. □ Suburban
    c. □ Rural
    d. □ Mixed
    e. □ Cannot tell/Not reported
    f. □ Other (Specify):
      Comments:
  14. Intervention delivery target? (pg. __)
    a. □ Group
    b. □ Individual
    c. □ Cannot Tell/Not Reported
    d. □ Other (Specify):
      Comments:
  15. Type(s) of Treatment: (pg. __)
    a. □ (insert a brief narrative description here – no more than one paragraph) —
    b. □ Select the method of delivery? (pg.)
      i. Face‐to‐face training (stand and deliver)
      ii. Counseling or mentoring
      iii. Online module
      iv. Written curriculum
      v. Other (Specify):
        Comments:
  16. Over what span of time was the intervention delivered to the treatment group? (pg. __)
    a. □ One session
    b. □ Within one week
    c. □ Within one month
    d. □ Between 1 month and 3 months
    e. □ Between 4 months and 6 months
    f. □ Between 7 months and 12 months
    g. □ More than one‐year
    h. □ Cannot tell/Not reported
  17. Length of each intervention treatment session? (pg. __)
    a. □ In minutes: __
    b. □ Cannot tell/Not reported
  18. Number of intervention treatment sessions/dosages? (pg. __)
    a. □ Number: __
    b. □ Cannot tell/Not reported
  19. Who delivered the intervention to the treatment group? (pg. __)
    a. □ Researcher
    b. □ Health care provider (e.g., physician, PT, OT, nurse, SLP, psychologist)
    c. □ Educator
    d. □ Service provider (e.g., counselor, employment specialist, VR counselor, program coordinator, disability support provider)
    e. □ Individual with disability
    f. □ Family member or caregiver
    g. □ Cannot tell/Not reported
    h. □ Other (Specify): handyman
      Comments:
D. **Outcome Tests/Measures**
  20. Norm Referenced Test(s) (pg.)
    Name:
  21. Non‐Norm Referenced Measure(s) (pg.)
    Name:
  22. Who administered the measure? (pg.)
    a. □ Researcher
    b. □ Health care provider (e.g., physician, PT, OT, SLP, psychologist)
    c. □ Educator
    d. □ Service provider (e.g., counselor, employment specialist, VR counselor, program coordinator)
    e. □ Peer
    f. □ Self‐administered
    g. □ Family member or caregiver
    h. □ Cannot tell/Not reported
    i. □ Other (Specify):
E. **Research Design**
  23. Design type? (pg. __)
    a. □ RCT
    b. □ Quasi‐Experimental
    c. □ Other (Specify):
  24. Recruitment pool? (i.e., individuals who were treated/received intervention) (pg. __)
    a. □ Criterion – given a pretest/checklist to determine eligibility
    b. □ Referral – obtained through another person
    c. □ Existing group – like a quasi‐experimental group – uses individuals within an existing group
    d. □ Volunteer – broad solicitation for participants through various outlets
    e. □ Cannot tell/Not reported
    f. □ Other (Specify):
  25. Subject assignment? (pg. __)
    a. □ Individual random
    b. □ Whole group random
    c. □ Individual matched‐random
    d. □ Non‐matched and non‐random
    e. □ Cannot tell/Not reported
    f. □ Other (Specify):
  26. Method of random assignment? (pg. __)
    a. □ Random number generation
    b. □ Coin flip
    c. □ Cannot tell/Not reported
    d. □ Other (Specify):
  27. Blinding?
    a. □ Researcher (pg.)
    b. □ Participants (pg.)
    c. □ Intervener (pg.)
    d. □ Assessor (pg. __)
    e. □ No blinding (pg.)
    f. □ Cannot tell/Not reported
    g. □ Other (Specify):
  28. Attrition? (pg. __)
    a. □ None
    b. □ % dropped out ‐ ____
    c. □ Cannot tell/Not reported
  29. Method of Analysis? (pg. __)
    a. □ Intention to treat – they tested the same number of individuals at pre‐ as at post‐test
    b. □ Treated participants only ‐ when the number reported on is different than the initial group
    c. □ Cannot tell/Not reported
F. **Outcome Information**
  30. What type of direct community access outcomes were measured? (pg.)
    These outcomes provide direct access to or participation in the community – can identify a place where it happens.
    a. □ Integrated competitive employment
    b. □ Continued learning, education, or training
    c. □ Community housing
    d. □ Civic involvement (e.g., voting,
    e. □ Increased recreation and leisure activities
    f. □ Navigating the community/accessing community
    g. □ Cannot tell/None reported
    h. □ Other (Specify):
  31. What type of related community outcomes were measured? (pg.2319)
    These outcomes are a related dimension of community participation – outcomes we know to be associated with community participation but are not directly in and of the community.
    a. □ Increased self‐determination, self‐efficacy, or self‐awareness
    b. □ Improved physical health
    c. □ Improved mental health
    d. □ Improved quality of life
    e. □ Increased family support/activities in the home
    f. □ Increased social networking
    g. □ Individualized goal attainment
    h. □ Increased service access or navigation skills
    i. □ Cannot tell/None reported
    j. □ Other (Specify):
G. **Results**
  32. Effect Size Data

**Table:**

Outcome name	Tx Pre Mean	Tx Pre SD	Tx Pre n	Tx Post Mean	Tx Post SD	Ctl Pre n	Ctl Pre Mean	Ctl Pre SD	Ctl Pre n	Ctl Post Mean	Ctl Post SD	Ctl Post n

**Table:**

Outcome name	p‐value	F‐test	t‐test
			
			
			
			
			
			
			
